# Night waking among breastfeeding mothers and infants: Conflict, congruence or both?

**DOI:** 10.1093/emph/eou006

**Published:** 2014-04-08

**Authors:** James J. McKenna

**Affiliations:** Department of Anthropology, University of Notre Dame, Notre Dame, IN 46556, USA

**Keywords:** infant night waking, maternal–infant conflict, mutual benefits, arousal functionality, maternal-induced infant arousals and breastfeeding

Thank you for the privilege of commenting on David Haig’s interesting and timely proposal ‘Troubled sleep: night waking, breastfeeding, and parent–offspring conflict’.

Haig’s main hypothesis, written quite superbly, is a simple one. He argues that frequent night waking to breastfeed poses a significant challenge to mothers sleep and is the infant’s way of prolonging lactational amenorrhea. Specifically, this has the effect of augmenting the inter-birth interval (IBI) essentially forestalling the infant from having to compete with a sibling for maternal resources. The author worries and makes the point several times that those like myself conducting evolutionary-based work on infant sleep conflates fitness and health. A central part of his thesis is that two well-documented genetic (partial or whole) deletions on Chromosome 15, associated with Prader Willi and Angleman Syndromes are the basis for proving a genomic conflict existing between a paternal gene (promoting infant wakening) and the maternal gene (on Chromosome 15) promoting infant sleep consolidation.

I find his essay very thought-provoking, especially how he perceives my own and others theoretical orientations who specifically investigate (from an evolutionary point of view) contemporary infant feeding and sleeping arrangements in western industrialized societies. I certainly benefited from his critique and was moved forward in my thinking by the theory he proposes. But I do not think that this perspective or its future application will necessarily trump what I think empirical research has demonstrated are critical factors, functions and interdependent physiological processes likely governed by multiple gene sets, that, I argue, likely serve as a better explanation of infant night wakening, and, thus, offer a both professionals and parents alike a better chance to manage and understand night waking *in all of its diverse forms.*

And what I mean by all their ‘diverse forms’ is that after observing hundreds of the circumstances by which infants awaken (in real time and from infra red videos), I have come to appreciate that it is not always the infant who is responsible for them, nor is there only one or two functions (at least in a proximate sense) that can be associated with them, either. These include (but are not necessarily limited to) how infant thermoregulation is effected by mother’s presence in turn effecting arousals and night waking, i.e. the warmer the environment the more infants awake. Moreover, infants and mothers are induced to awaken by an arousal exhibited by the other (within seconds) therein creating inter-connected, mutually dependent, synchronous arousals [1, 2].

Infant waking can be related to breathing patterns, too. For example, apneas are distributed differentially across sleep states which can induce night waking in those stages in which the infants have the most (stages 1–2 especially) when mothers and infants bedshare [3]. In turn, these protective awakenings following an apnea often lead to a *temporally related* breastfeed. Heart rate and oxygenation (likewise) change by way *maternally induced*, intermittent, spontaneous nighttime events such as a maternal cough or abrupt movement, entwining the sensory modalities of both the mother and infant simultaneously.

I am dancing around the idea that night waking may have evolved to fulfill and accommodate perhaps what Myron Hofer [4] calls ‘hidden regulatory effects’, a cornerstone of much of my research, which can carry with them ‘hidden’ benefits. The beauty of observing infant wakings either in the home or in the laboratory using electrophysiological measuring techniques is that the behavior can be seen to be intimately linked to underlying EEGs, breathing patterns, changes in sleep architecture, body temperature and linked simultaneously to maternal physiological and behavioral events [5].

Maybe what is really important for infant well-being (just to push the point a bit) and eventually leaving more descendants, to use the author’s words, is how sensitive the infant is to arousing in the first place, or how conditioned they are to BE sensitive to what their mother is doing such as by responding in parallel to her, and in synchrony with mother’s own arousals. By being sensitive, which leads to more waking infants spend more time in safer sleep, i.e. light sleep, Stages 1–2, and not in deeper, adult like sleep (Stages 3–4) which because of its higher arousal threshold is more difficult for an infant to get awake from, in order to terminate a life-threatening apnea [6]. The more the mother induces arousals in her infant, which she does all night the more the infant feeds. In fact 40% of infant arousals in our study were caused by the mother having aroused 2 s before the infant aroused, and a good number of these maternal-induced wakening led to a feeding that might not have occurred had mother not induced it. (Incidentally, 60% of maternal arousals are explained by the infant having aroused ±2 s before her). Such data quickly move any discussion of ‘origins’ either in the proximate or ultimate sense to the role that mothers play in this context [1, 2].

What I am speculating about is that perhaps mothers evolved to induce arousals (to have her baby feed) because lots of arousals for several reasons increase an infant’s chance for survival, as well as help protect mothers from a variety of diseases not the least among them are breast and ovarian cancers. As regards infant fitness, we know that babies that die of Sudden Infant Death Syndrome (SIDS) likely had some kind of arousal deficiency (did not arouse sufficiently well enough) due to serotonergic brainstem abnormalities [7] that could be mediated, we hypothesized, by practice *vis-a-vis* externally induced maternal arousals [1, 2].

Externally induced arousals, yet, again could function to improve awakening skills (practice makes perfect) which is hardly a benign talent since infantile cardio-pulmonary perturbations are corrected by the infant arousing from sleep that leads to oxygenation. Arousing is an infant’s best defense [1, 2, 6] against a range of potential physiological challenges.

And, also as we have shown that it is not just the fact that infants wake to feed, they wake for non-nutritive purposes, too, to be cuddled and to be touched, though attributing what purpose it serves is difficult. In other words, infants are not always waking for food but, to be (quite possibly) reassured emotionally and when they awaken and subsequently mothers touch, hug, inspect or whisper to them we witness on our monitoring screens a suite of physiological changes including increased heart rate [8] and higher oxygen levels measured by oximetry, all of which is remarkable to observe [9, 10].

Mother's odors and milk olfactory cues likewise seem to keep infants ‘ready to arouse’ and to wake, too, though we cannot measure this, but we have noted and quantified body orientation and in what direction the infant is facing while asleep which is toward the mother for almost 100% of the night!

It is perhaps also important to note in appreciating the point that infant night wakenings are a heterogeneous phenomenon that even among exclusively breastfeeding mothers the number of times infant awake to feed varies enormously from infant to infant. Some babies wake a couple of times a night while others 13–15 times though mothers generally only recall in the morning 40–50% of them, if bedsharing [11].

Night wakening also greatly depends on EXACTLY how close the mother actually is to the infant. We were surprised that with the infant sleeping just 10 or so feet away the number of breastfeeds (compared with when they were bedsharing) could drop as much as 50–70% [11]. And Ball has shown likewise that in-bed, side car (infant sleeping in a bassinet attached to bed) and the infant sleeping in a stand-alone crib some 5 or 6 feet away from mother showed a remarkable almost exponential diminution of breastfeeding frequency based on degrees of proximity [12].

Likewise, night waking is often determined and/or regulated by daytime breastfeeding frequencies, too. For example, for various reasons, some infants are heavy night feeders, while some can be daytime heavy feeders, some are neither but the feeds are more evenly distributed 24/7, which is and probably always has been determined in part by maternal activity and agency, including work schedules and also by way of changing growth, metabolic and developmental caloric requirements, and likely by the infants own personality characteristics. So the point here is that growth or metabolic requirements of different babies, and their mothers willingness and abilities to meet those needs must be seen over a 24-h basis if we are to understand in detail how or if breastfeeding promotes a longer inter-birth interval.

I guess Professor Haig might say that those of us doing this work surely focus and address more proximate mechanisms but insofar as some or many of these awakenings potentially influence the infants health, survival and, thus, leaving descendants (here is this sticky issue again). It seems difficult sometimes to keep them clearly separated and I am not entirely sure I know what to conclude here.

But (and I return to this issue) not all night waking is the same. The question is how do we sort out the contributions these mutually asserting factors make, to disentangle function or outcome within what amounts to be a taxonomy of ‘TYPES’ of waking, and does this diversity challenge the conflict model as proposed and if so how.

I was a little less clear about how to respond to his point about casomorphins, which are equally present in bovine milk having the same soporiphic effect but with one caveat: the cows milk source increases the risk of stimulating abnormal inflammatory responses in the human infant gut with the soporiphic constituent being tryptophan. As regards gut motility in general distinctions should be made between gastric emptying and intestinal transit time. Gastric emptying is primarily a function of the fat content of the milk (approximately equal in bovine, human and formula) while casein which is in excess in cows milk and forms a curd that delays gastric emptying, as Haig implies. But it might be helpful also to recall that intestinal motility is primarily a function of the amount of lactose which is higher in content in human breastmilk which explains why compared with Bovine milk breastfeeding infants have shorter transit time.

Obviously the theoretical speculations and conjectures the author makes here would be strengthened if it could be demonstrated that what he describes (or what is possible) by way of these paternal and maternal genes actually can be shown and or quantified. I think there is a major leap of faith one has to take here. That is, can it be empirically demonstrated that among these children effected by these terribly debilitating syndromes that the paternal gene really does promote waking, and that the maternal gene really does promote sleep consolidation? I ask this seriously because after reading several review articles of these syndromes it seems that there is enormous variation in how each of these syndromes express themselves, individual to individual, as their exists a particularly wide range of deficiencies in which the form and degree and severity across effected children find expression in enormously different ways. Is there any data available on these children that really look to see if this patterns hold? It is also problematic perhaps, to infer normative function of genes from pathology?

Moreover, I note that the author dismisses (if I interpret his point correctly) any substantial role that culture might play in thinking about or determining trade-offs including access to calories or degrees of conflict and costs in the larger sense, but I cannot altogether ignore how human beings embody beliefs and expectations (environmental conditions, obviously) intrinsic to or dependent on, our respective cultural values and ideologies—how, in this case, we interpret and assess, our infants sleep behavior as it can and does influence how we experience it, in a physical way. The cultural context within which infant sleep develops, I think, should, as well as changing material conditions, be reckoned in as regards ultimate costs and effects of night waking, at least as perceived by mothers given that perception is not benign.

I am thinking here (as indirect evidence) of Jordan’s [13] analysis of birth in four different cultures where pain levels and the overall duration of labor and the birth experience altogether of women are all influenced by what giving birth meant and what women came to think about it and what they expected their experience to be like, based on what the cultural ‘take’ on birth was. For example, in the USA birth is seen as a painful and dangerous medical emergency while in the Netherlands, to pick one example, it is just a ‘normal part of life’.

Compared with the rest of the world, our parents *do* carry a huge amount of baggage about how terrible it will be dealing with an infant’s sleep. Indeed, we are taught to expect an adversarial relationship with our babies (as regards sleep) even before we meet them [14]. Due to placing infants at odds with their emotions, i.e. socially isolating them for sleep, and/or minimizing contact which is exactly what infants seek and need it is no surprise that western parents (surely the most well read and informed) nonetheless remain the most obsessed, judgmental, disappointed, exhausted and the least satisfied parents on the planet! I attribute much of this to the fact that in the western traditional infant sleep models and recommendations and conceptual expectations have always heretofore been determined by social ideologies, social ‘wish lists’, having little to do with who babies are biologically, preferring instead to define infant needs in terms of who we want them to become and notions as to how, for example, to make them ‘independent’ at young ages [14] and, thus, we create the very sleep environments and unrealistic parental expectations that create and perpetuate the very sleep ‘problems’ sleep ‘experts’ are asked to solve [15]. Indeed, I would have guessed Professor Haig might have appreciated the critique my colleagues and I have made against the current models of infant sleep because, and afterall, it really is not nice nor maybe even possible to fool mother nature, which is the same kind of theoretical orientation he adopts in his own work.

Surely, the authors cultural experience and developmental milieu and participation bring great salience to the notion of parent–infant sleep struggles because of the culture in which he lives, struggles that are as far as the ethnographic data reveal *not* experienced by the majority human beings for which questions about infant sleep are for the most part never asked, questions like what is normal, where should an infant sleep, in what position (back, belly or side) should they sleep, how should they feed, or are you a ‘good’ parent, do you have a ‘good’ baby that ‘sleeps through the night’ or, how long should you breastfeed and so on.

What I am saying is I cannot agree that cultural and developmental processes unique to the industrialized west are not biasing all of us toward thinking of infant sleep as being inherently more difficult than it evolved to be, partially explaining why a notion of ‘troubles’ would find itself very prominently, first and foremost, in the title of Haig’s paper, as opposed to what might be found on a paper dealing with infant night waking in China, Japan, Vietnam, India or the Philippines to name but a few diverse cultures where very different assumptions, attitudes, expectations and, hence, parental experiences about infant sleep can be found [15].

It is obvious that I find myself mostly uncomfortable with the conceptual/theoretical notion that night waking depends on a single gene hypothesized to be the basis of human infant night waking—and it is assumed to be an evolved trait rather than as a complex of evolving *transacting* traits. My research leads me to say this is all too simple, as I see night waking as a system of traits which cannot altogether be seen devoid of potentially important cultural influences and context, too. And while I surely think it is possible for increased or exuberant sucking and increased contact with mothers nipple day AND night can prolong the IBI I do not think that night waking can be used to explain it sufficiently well enough, if at all, when seen alongside the more immediate multiple, transactional purposes and causes that these diverse types of night wakening serve or reflect.

Theoretically, I guess I hark back to George Gaylord Simpson’s, *The Meaning of Evolution* (now here is an oldie), who warned us not altogether to confuse a potential functional explanation for an understanding of its origin insofar as what behaviors might do cannot necessarily provide the explanation of how and why they emerged, or what they *actually* do, at least originally. And this difference of opinion here might well reflect differences that emerge when primary empirical observations and contemporary (diverse) physiological data are used in lieu of theoretical, inferential methods of analysis.

I realize this view of mine may also reflect perhaps my emphasis or adherence to of a more integrative anthropology that looks to the confluence of different lines of research reflecting some larger theoretical fissures or paradigmatic differences in ‘world views’ between Professor Haig and myself. Regardless, I do not or would not discount the potential validity of the author’s view based on how I prefer to understand how evolution works altogether.

Professor Haig is right in noting the problem with my 1993 statement in an article published in *Sleep* [16]. ‘Infant needs, and parental responses to those needs, constitute a dynamic, co-evolving interdependent system shaped and designed by natural selection to maximize the chances of infant survival and, hence, parental reproductive success’. I stand corrected. I agree with him that evolution will not always or generally ‘maximize’ infant survival (since as he points out and I agree that there are significant trade-offs) and, furthermore, maximizing *each* infant’s survival does *not* necessarily translate to maximized parental lifetime reproductive success. I appreciate having to think about why my statement is untrue. At very least we should have used the word ‘promote’ instead of ‘maximize’, no doubt.

Still, in reflecting on what the author finds problematic with my theoretical orientation as I study infant sleep I am reminded of an old TV commercial from the 60s. It involved an argument between two young women as to whether a mint was a breath or a candy mint. It went something like this: one young women during the commercial says to another ‘Certs is a “candy mint”,’ while the other counters with: ‘No, Certs is breath mint’. And then suddenly a man’s authoritative voice chimes in with the words flashing on the screen in BIG letters as he bellows ‘STOP, you ‘re both right!’

What I am saying is I am not sure my critique or perspective negates Haig’s perspective so much as complicates it? I am not sure that we are not both right to degrees and that these approaches are mutually exclusive. Indeed, as long as a behavior initially does not reduce fitness, it can certainly prove beneficial, of course, rather opportunistically (another of Simpson’s themes) ultimately enhancing the behaviors original functional contribution, perhaps even replacing it. Surely it would seem that if an infant can increase the time it has to secure its own needs before another infant comes along, then surely there is added value or benefit here.

I also think that by using quotes (several of mine) that had a larger context to them over simplifies my intentions and creates a kind of straw man argument. The author implies that evolutionary pediatrics, a phrase I try not to use, is committed to some over-arching allegiance to, or belief in, an idealized Pleistocene, some Bowlby-like fantasy EEA to which the author makes comedic reference. Quotes are used to suggest that Professor Helen Ball and myself believe that all we twenty-first century people have to do is to return to the Pleistocene way of life, to go back to a ‘natural’ way to care for our babies and all will be well with the world. I do not think this way at all, although admittedly I did appreciate his clever metaphoric writing about it, i.e. in using the Garden of Eden and the ‘mean—old—serpent’ to represent the inherent mother–infant conflict, he sees lurking there, a serpent he suggests Professor Helen Ball and I are blind to. While this characterization does not reflect how, why and what justification I use theoretically I think I need to be more careful and improve my own rhetorical strategy. So assume some responsibility for Professor’s Haig’s impression here.

Perhaps the author will be interested to hear that both Professor Helen Ball and myself have recently written independently critiques of the traditional notion of the EEA and what can and cannot be useful from Bowlby’s work [17, 18].

Also, both of us incorporate the concept of trade-offs and life-history theory as a way to interpret what we often see in our mother–infant studies. Indeed, I was a co-pi in a 5-year NIH backed project at the University of Notre Dame Mother Baby Behavioral Sleep Laboratory that examined the ways in which young, first time, ‘at risk ‘moms prioritize risks and exhibit trade-offs as regards their nighttime infant care practices, given their divergent resources and circumstances. Both of us are very much aware of and have documented how and when mother–infant conflict finds expression [19, 20, 21].

More importantly, he delivers a subtle but important warning with which I agree, wholeheartedly and work hard to promote, and that is to avoid simplistic sweeping public health recommendations emerging from our work or any work. I may be mistaken but he seems to imply that this is what has occurred. Yet, although I do support and argue in my articles and publicly for informed parental choices, and that at very least, and if possible mothers should consider exclusively breastfeeding and never leave an infant to sleep outside parental supervision (in separate rooms). I avoid generic recommendations as to what any particular family should do. This is not the same as recommending that all babies should bedshare (or never bedshare) as bedsharing safety is determined specifically by how it is practiced and by whom and what adverse factors could be associated with it and how much knowledge families have access to concerning risks and benefits. Since I do not know the circumstances by which parents live such specific recommendations are inappropriate if not dangerous.

The difficulties of translations (from research to public health recommendations) of what we learn are quite obvious and have been addressed in multiple papers of mine [22–25]. In fact I am one of the major critics of the tendency by medical authorities to reduce complex issues such as bedsharing and infant care practices to simple one-size-must-fit-all, sweeping generalizations. This has placed infants in harms way in the past and it will continue to be the case, if history means anything at all.

Haig comes down very harshly on those of us who have specifically challenged (and continue to) the tenacious, over-reaching claims and recommendations made by the pediatric sleep research community as regards what constitutes healthy, normal and safe-optimal infant sleep (for all infants). And perhaps he is right in implying that it is our fault that we have not made it more clear that anthropologists studying infant sleep remain quite aware of how many western infant lives have been saved by current medical research and practice, at least by research emerging within other pediatric domains. But that said, recall that even after scores of refereed papers and new research showing its deficiencies and serious limitations published in the top pediatric and sleep research journals, studying the bottle-fed, solitary sleeping infant in a sleep laboratory remains the gold standard method used to derive species-wide data on what is still assumed by pediatric sleep researchers to constitutes ‘normal’ *human* infant sleep behavior and physiology, despite the fact that 77% of American mothers now breastfeed their infants which alone invalidates the utility of developmental models of infant sleep derived exclusively from the solitary sleeping, bottle fed infants. And I cannot forget (and what has driven my own career, to add a personal note) is knowledge that it was the cultural dismantling of the three, biologically interdependent components of species-wide, ‘normal’ human infant sleep that ultimately led to the tragic deaths possibly of as many as 400 000 western infants. To be specific, driven by cultural ideologies (beliefs in nighttime privacy for parents, the importance of infant sleep consolidation as early in life as possible and autonomy and separation for healthy infant development) western societies shifted from social (forms of co-sleeping) to solitary separate sleep, from breastmilk and breastfeeding to formula or cows’ milk, bottle-feeding and from the safe back-sleeping position (required naturally if breastfeeding) to stomach-prone sleep to promote deeper sleep (uninterrupted sleep) and each one of these changes proved to be independent risk factors for SIDS and or SUDI!

But, just to clarify, what I do argue is that the confluence of evolutionary, cross-cultural and cross species data serve as a powerful *beginning* point to ask important questions never asked as regards infant sleep and sleeping arrangements in western industrialized cultures. But evolutionary-based research ideas and findings *cannot* be the end point in looking for ways to use what is learned. I think if anything anthropologists are more aware of how complex and tricky making sweeping recommendations can be when intended for a broad spectrum of people [22]. What we do argue is that parents should be empowered to make informed decisions for themselves and not to have medical authorities filtering what *they think* parents need to know, denying them, for example, knowledge that can minimize risks should they choose, for example, to sleep in bed with their infants, which is a choice only theirs to make. I wish that the very insular, seven person committee of the American Academy of Pediatrics who limited what evidence they used to label any and all mother–infant bedsharing as ‘hazardous’ could become more sensitive to this critical issue, and come to appreciate the many legitimate reasons why mothers and fathers choose to bedshare [26]. I have always agreed with H.L. Mencken who wrote ‘For every complex problem, there is one, simple, wrong solution.’

One final point perhaps a bit more tangential (and I apologize for making this so long), breastfeeding benefits have proved to be dose-specific, as I mentioned earlier, the more babies feed the stronger the protection from all kinds of health challenges. In order to feed obviously they must awaken. Consider that even in a highly sanitized county such as our own, the USA, where infectious load is relatively low the importance of breastfeeding to infant survival, as it turns out, is very significant and until recently here in the USA its survival value was underestimated. It was not until Chen and Rogan established through their epidemiological study that between approximately 720 western infants die each year specifically because they were not breastfed [27].

Realizing that genetic disorders are weak evidence for the adaptive function of the imprinted DNA, especially since those disorders are massive, and give little insight into the normal function of those stretches of DNA, and figuring out a significant place in his hypothesis for all of these very real other benefits protections for both the mother and infant associated with infant wakening (to breastfeed) constitute the main challenges to Professor Haig’s viewpoint.

## FUNDING

National Institutes of Child health and Human Development (NICHD) R0! Grant Number 27482 and by a Shannon Award made by the National Instiutes of Health.

**Conflict of interest**: None declared.

## References

[1] Mosko S, Richard C, McKenna J (1997). Infant arousals in the bedsharing environment: implications for infant sleep development and SIDS. Pediatrics.

[2] Mosko S, Richard C, McKenna J (1997). Maternal sleep and arousals during bedsharing with infants. Sleep.

[3] Richard C, Mosko SS, McKenna JJ (1998). Apnea and periodic breathing in bedsharing and solitary sleeping infants. J Appl Physiol.

[4] Hofer M, Gubernick D, Klopfer P (1981). Parental contributions to the development of offspring. Parental Care in Mammals.

[5] McKenna JJ (1994). Co-sleeping mothers and infants influence each other’s sleep physiology: overview and implications for SIDS. J Dev Physiol.

[6] Mosko S, Richard C, McKenna J (1994). Infant sleep architecture during bedsharing and possible implications for SIDS.

[7] Kinney HC, Myers MM, Belliveau RA (2005). Subtle autonomic and respiratory dysfunction in sudden infant death syndrome associated with serotonergic brainstem abnormalities: a case report. J Neuropathol Exp Neurol.

[8] Mosko SS, Richard C (2004). Mother–infant bedsharing is associated with an increase in heart rate. Sleep.

[9] McKenna JJ, Mosko SS, Richard C (1994). Experimental studies of infant parent co-sleeping: mutual physiological and behavioral influences and their relevance to SIDS (sudden infant death syndrome). Early Hum Dev.

[10] McKenna J, Mosko S (1994). Sleep and arousal, synchrony and independence among mothers and infants sleeping apart and together (same bed): an experiment in evolutionary medicine. Acta Pediatr Int J Pediatr Suppl.

[11] McKenna J, Mosko S, Richard C (1997). Bedsharing promotes breastfeeding. Pediatrics.

[12] Ball HL, Ward-Platt MP, Heslop E (2006). Randomised trial of mother–infant sleep proximity on the post-natal ward: implications for breastfeeding initiation and infant safety. Arch Dis Child.

[13] Jordan B (1978). Birth in Four Cultures: A Cross-Cultural Investigation of Childbirth in Yucatan, Holland, Sweden and the United States.

[14] Schaefer C, DiGeronimo TF (1992). Winning Bedtime Battles: How to Help Your Child Develop Good Sleep Habits.

[15] McKenna JJ, Gettler LT, Loughlin G, Carroll J, Marcus C (2008). Cultural influences on infant sleep biology and the science that studies it: toward a more inclusive paradigm, part II. Sleep and Breathing in Children: A Developmental Approach.

[16] McKenna JJ, Thoman E, Anders T (1993). Infant–parent co-sleeping in evolutionary perspective: imperatives for understanding infant sleep development and SIDS. Sleep.

[17] Ball H, Russell C, Narvaez D, Panksepp J, Schore A (2012). An evolutionary perspective on breastfeeding and sleep. Evolution, Early Experience and Human Development: From Research to Practice and Policy.

[18] McKenna JJ, Gettler LT, Narvaez D, Panksepp J, Schore A (2012). It’s dangerous to be an infant: on-going relevance of John Bowlby’s environment of evolutionary adaptedness (the EEA) in promoting healthier births, safer maternal-infant sleep, and breastfeeding in a contemporary western industrial context. Evolution, Early Experience and Human Development: From Research to Practice and Policy.

[19] McKenna JJ, Volpe L, Wetherall W (2006). Longitudinal study of beliefs, sleeping arrangements, feeding practices and attachment of at risk teen moms compared with high and low risk older control moms: implications for SIDS. Pediatr Infant Health J Can Paediatr Soc.

[20] McKenna J (2006). Assessing trade-offs between potential benefits and risks of increased nighttime contact between mothers and infants. Pediatr Infant Health J Can Paediatr Soc.

[21] Volpe L, Ball H, McKenna JJ (2013). Nighttime parenting strategies and sleep related risks to infants. Soc Sci Med.

[22] Gettler LT, McKenna JJ (2010). Never sleep with baby? Or keep me close but keep me safe: eliminating inappropriate ‘safe infant sleep’ rhetoric in the United States. Curr Pediatr Rev.

[23] McKenna JJ, Ball H (2010). Early infant sleep consolidation is unnecessary barrier to breastfeeding. E-Pediatrics.

[24] McKenna JJ, Ball H, Gettler LT (2007). Mother–infant co-sleeping, breastfeeding and sudden infant death syndrome: what biological anthropology has discovered about normal infant sleep and pediatric sleep medicine. Yrbk Phys Anthropol.

[25] McKenna JJ, McDade T (2005). Why babies should never sleep alone: a review of the co-sleeping controversy in Relationship to SIDS, breast feeding and bedsharing. Paediatr Respir Rev.

[26] McKenna JJ, Volpe L (2007). An internet based study of infant sleeping arrangements and parental perceptions. Infant and Child Development.

[27] Chen A, Rogan WJ (2004). Breastfeeding and the risk of postnatal death in the United States. Pediatrics.

